# A study on robot force control based on the GMM/GMR algorithm fusing different compensation strategies

**DOI:** 10.3389/fnbot.2024.1290853

**Published:** 2024-01-29

**Authors:** Meng Xiao, Xuefei Zhang, Tie Zhang, Shouyan Chen, Yanbiao Zou, Wen Wu

**Affiliations:** ^1^Department of Rehabilitation, Zhujiang Hospital, Southern Medical University, Guangzhou, China; ^2^School of Mechanical and Automotive Engineering, South China University of Technology, Guangzhou, China; ^3^School of Mechanical and Engineering, Guangzhou University, Guangzhou, China; ^4^Rehabilitation Medical School, Southern Medical University, Guangzhou, China

**Keywords:** robot force control, impedance control, reinforcement learning, deep Q-network (DQN), Gaussian mixture model/Gaussian mixture regression (GMM/GMR)

## Abstract

To address traditional impedance control methods' difficulty with obtaining stable forces during robot-skin contact, a force control based on the Gaussian mixture model/Gaussian mixture regression (GMM/GMR) algorithm fusing different compensation strategies is proposed. The contact relationship between a robot end effector and human skin is established through an impedance control model. To allow the robot to adapt to flexible skin environments, reinforcement learning algorithms and a strategy based on the skin mechanics model compensate for the impedance control strategy. Two different environment dynamics models for reinforcement learning that can be trained offline are proposed to quickly obtain reinforcement learning strategies. Three different compensation strategies are fused based on the GMM/GMR algorithm, exploiting the online calculation of physical models and offline strategies of reinforcement learning, which can improve the robustness and versatility of the algorithm when adapting to different skin environments. The experimental results show that the contact force obtained by the robot force control based on the GMM/GMR algorithm fusing different compensation strategies is relatively stable. It has better versatility than impedance control, and the force error is within ~±0.2 N.

## 1 Introduction

The applications of robot-skin contact are diverse, including uses in robotic medical-aided diagnosis, massage, aesthetic nursing, and other scenarios (Christoforou et al., 2020). In these scenarios, robots can work continuously without rest, and simultaneously, they can maintain highly consistent movements, strength, and speed, so they can partially replace human labor (Kerautret et al., 2020). Good robot force control is essential for efficient and comfortable robot-skin contact experiences. Robot force control requirements must address safety, precision, and variability; if the robot applies too little force, it may fail to achieve the intended effect, and if it applies excessive force, it may cause skin pain or injury. The biological characteristics of the skin determine differences in the mechanical characteristics of the skin of different individuals (Zhu et al., 2021); therefore, the robot usually faces unknown contact environments. Ensuring the accuracy of robot interaction considering the characteristics of different people's skin is the focus of current research.

Many researchers and institutions have studied robot force strategies, and impedance control plays an important role in these strategies. Impedance control constructs a contact model between a robot and human skin and flexibly changes dynamic characteristics during interactive tasks (Jutinico et al., 2017). Some scholars, such as Li S. et al. (2017) and Sheng et al. (2021) conducted experimental research on the contact process between the robot and skin based on impedance control. The control parameters of impedance control, such as stiffness and damping, require utilizing manual adjustment or trial and error, and the controller is insensitive to the uncertainty of the external environment. To adapt robots to the flexible environment of human skin, other scholars, such as Liu et al. (2021), Khoramshahi et al. (2020), Li et al. (2020), Ishikura et al. (2023), Huang et al. (2015), and Stephens et al. (2019) used adaptive algorithms and intelligent algorithms for optimizing the impedance control parameters. The skin, being a living tissue, has biomechanical properties, such as elasticity, viscoelasticity, non-linearity, and anisotropy (Joodaki and Panzer, 2018). The mechanical characteristics of the flexible contact environment faced by the robot are often dynamic, and traditional force controllers cannot explore unknown environments.

Reinforcement learning can be used to explore control strategies in robots. Through reinforcement learning, robots can learn how to adjust their control strategies to perform better and adapt to external environmental changes by interacting with that environment (Suomalainen et al., 2022). Many scholars have used reinforcement learning to explore the optimal control strategy; for example, Luo et al. (2021) proposed a method based on Q-learning to optimize online stiffness and damping parameters. Ding et al. (2023) used reinforcement learning to analyze and optimize the impedance parameters. Bogdanovic et al. (2020) used a deep deterministic policy gradient to learn the robot output impedance strategy and the required position in the joint space. Meng et al. (2021) adaptively adjusted the inertia, damping, and stiffness parameters through the proximal policy optimization algorithm. These reinforcement learning algorithms have good versatility and self-adaptability in the interaction process and perform well in the simulation environment, but when used in practical applications, they must often address multiple interactions. Therefore, some scholars have begun using the model-based method to reduce the number of actual interactions and improve the utilization rate of the algorithm (Hou et al., 2020). For example, Zhao et al. (2022) proposed a model-based actor-critic learning algorithm to safely learn strategy and optimize the impedance control. Anand et al. (2022) used a model-based reinforcement learning algorithm, which integrates probabilistic inference for learning force control and motion tracking. Roveda et al. (2020) proposed a variable impedance controller with model-based reinforcement learning, and Li Z. et al. (2017) identified adaptive impedance parameters based on the linear quadratic regulator. In most of the aforementioned studies, the contact environments are rigid, and the established models are relatively stable. These models can predict the dynamic evolution of the environment and the generation of rewards. Furthermore, reinforcement learning agents can identify and make better decisions, so the quality and accuracy of the model directly affect the performance results of reinforcement learning. While the contact between the robot and human skin is flexible, this environment is more uncertain than the rigid environment, and using reinforcement learning to quickly and efficiently find the optimal strategy in practice has not been achieved (Weng et al., 2020).

Compared to traditional control for robot massage, the main contributions of this work are as follows.

(1) A robot force controller based on the Gaussian mixture model/Gaussian mixture regression (GMM/GMR) algorithm fusing different compensation strategies is proposed, which combines a traditional robot force controller and reinforcement learning algorithm.
(2) Two environmental dynamics models of reinforcement learning are constructed to simulate the contact process between the robot and the skin. The number of actual interactions of the reinforcement learning is reduced. At the same time, the practicability of the reinforcement learning algorithm is improved.
(3) The GMM/GMR algorithm fuses online and offline compensation strategies to improve the robustness and versatility of the algorithm and to adapt to different skin environments.

The remainder of the paper is structured as follows: in the second section, the impedance control strategy is constructed in the contact process of the robot. In the third section, two robot force control compensation strategies based on a deep Q-network (DQN) with dynamic models are proposed, and the strategy of reinforcement learning is learned offline. In the fourth section, an online compensation strategy is built based on a skin mechanics model. In the fifth and sixth sections, the experimental platform is built and experiments are conducted to verify the feasibility of the algorithm. A list of variables used in the paper are shown in Table 1.

**Table 1:** List of variables used in the paper.

**Placement**	**Variable**	**Description**
Impedance control	*m_*d*_*	Inertia parameter of impedance control
	*b_*d*_*	Damping parameter of impedance control
	*k_*d*_*	Stiffness parameter of impedance control
	Δẍ	Acceleration of the robot end-effector
	Δẋ	Velocity of the robot end-effector
	Δ*x*	Offset displacement of the robot end-effector
	*f_*r*_*	Reference contact force
	*f_*e*_*	Actual contact force
	*k*	*k-*th sampling period
	*T_*s*_*	Sampling period
	*e*	Difference between reference force and actual force
DQN	*s*	State
	*a*	Robot action, i.e., robot offset displacement
	τ	Trajectory of reinforcement learning
	*r*	Reward
	ė_*t*_	Change of the force error
	*R*	Discounted return
	γ	Discount factor
	π^*^	Optimal strategy
	*Q*(*s, a*, θ^−^)	Target value deep neural network
	*Q(s, a, θ*)	Predicted value deep neural network
	*L*	Loss function
	*y*	Value of the target network
	*G*	Experience samples
	*N*	Training iterations
	*Z*	Net activation value
	*U* ^l^	Activation value
	*b^*l*^*	Bias of the *l-*th layer
	*W^*l*^*	Weight of the *l-*th layer
	φ	Activation function, the ReLU activation function is selected.
	*δ^*l*^*	Error term for the *l*-th layer
	α	Learning rate
	λ	Regularization coefficient
BP neural network dynamics model	*NeT1.W*	Weight in the BP neural network
	*NeT1.b*	Bias in the BP neural network
	φ	BP neural network
	*a* ^1^	Compensation displacement obtained by DQN with BP neural network dynamics model
LSTM neural network dynamics model	ϕ	LSTM neural network
	*NeT2.W*	Weight in the LSTM neural network
	*NeT2.b*	Bias parameters in the LSTM neural network
	*C_*t*_*	Memory state
	*o*_t_	Output gate
	*i*_t_	Input gate
	*f*_t_	Forget gate
	*X_*t*_*	Input at the current moment
	*Net*2.*W_*f*_*,	Weights of the forget gate
	*Net*2.*W_*i*_*,	Weights of the input gate
	*Net*2.*W_*c*_*	Weights of estimated state
	*Net*2.*W_*o*_*	Weights of output gate
	⊙	Hadamard product
	σ	Logistic function with an output interval
	*H_*t*_*	Hidden state
	*a* ^2^	Compensation displacement obtained by DQN with LSTM neural network dynamics model
Skin mechanics model	*f_*s*_*	Force generated by skin deformation
	*x*	Coordinate of the robot when it is deformed
	*x_*e*_*	Initial coordinates of the skin
	*k_*s*_*	Elasticity coefficients
	*b_*s*_*	Damping coefficients
	*u^*s*^*	Compensation displacement based on displacement compensation with skin mechanics model
GMM/GMR algorithm	*t*	Time information
	*n*	Number of samples
	*u*	Represents the three kinds of compensation displacements
	*P*(*t, u*)	Joint probability distribution
	*M*	Number of Gaussian components in the GMM
	*π_*m*_*	Prior probability of the *m*-th Gaussian component
	μ_*m*_	Mean of the *m*-th Gaussian component
	Σ_*m*_	Covariance of the *m*-th Gaussian component
	*t* ^*^	The predicted time
	*u* ^*^	Predicted compensation displacement
	*u_*f*_*	Central distribution of/, final fusion strategy

## 2 Robot force control based on impedance control

In robot-skin interaction scenarios, the robot end-effector is equipped with a probe, which makes skin contact and moves along a set trajectory, and the force signal is collected through the sensor between the robot and the probe. To ensure safety during the contact process, the reference force of the contact force must be set and a force controller must be used to adjust the contact state of the robot and ensure that the robot follows the reference force. Impedance control can be used to ensure reasonable contact between robots and human skin; it simplifies the contact model between the robot and the human into a linear second-order system contact model with inertia, damping, and stiffness characteristics. The contact model adjusts the robot displacement based on the difference between the actual measured force and the reference force, while the characteristics of the contact model are adjusted using the inertia, damping, and stiffness parameters (Song et al., 2017). In the Cartesian coordinate system, in the normal direction of the contact between the robot and the skin, analysis is performed from only one dimension, and the position and contact force of the robot meet the following conditions (Li et al., 2018):


(1) $$\begin{array}{l} m_{d}\Delta ẍ+b_{d}\Delta ẋ+k_{d}\Delta x=f_{r}-f_{e} \end{array}$$

where *m*_*d*_, *b*_*d*_, and *k*_*d*_ are the inertia, damping, and stiffness parameters of impedance control, respectively; Δẍ, Δẋ, and Δ*x* are the acceleration, velocity and offset displacement of the robot end-effector, respectively; *f*_*r*_ is the reference contact force; and *f*_*e*_ is the actual contact force, which obtained after filtering. In the actual sampling system, the difference can be calculated as follows (Song et al., 2019):


(2) $$\begin{array}{l} \Delta ẋ\left(k\right)=\frac{\Delta x\left(k\right)-\Delta x\left(k-1\right)}{T_{s}}\\ \Delta ẍ\left(k\right)=\frac{\Delta ẋ\left(k\right)-\Delta ẋ\left(k-1\right)}{T_{s}} \end{array}$$

where *k* is used to represent the *k-*th sampling period, and *T*_*s*_ represents the sampling period. Substituting Equation 2 into Equation 1, can be calculated online as


(3) $$\begin{array}{l} \Delta x(k)=\frac{{eT_{s}}^{2}+b_{d}T_{s}\Delta x(k-1)+m_{d}(2\Delta x(k-1)-\Delta x(k-2))}{m_{d}+b_{d}T_{s}+k_{d}{T_{s}}^{2}} \end{array}$$

where *e* = *f*_*r*_ − *f*_*e*_. If the parameters of the contact environment are well-defined, the contact force can be well-tuned by selecting appropriate impedance parameters. However, the skin environment is usually unknown, and simply maintaining target impedance parameters does not guarantee a well-controlled contact force.

**Figure 1:**
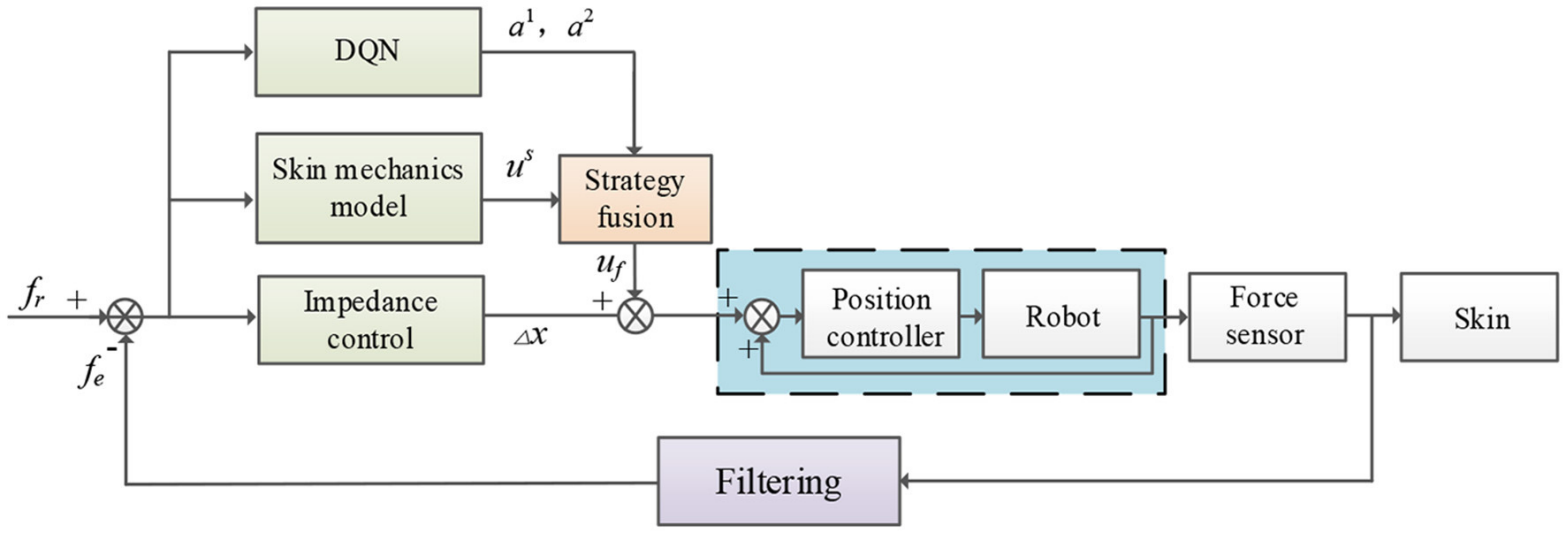
Flow chart of robot force control.

Therefore, a robot force control algorithm is proposed to compensate for the offset displacement of the robot Δ*x*(*k*). A deep reinforcement learning algorithm and a traditional compensation algorithm based on a physical model of the skin are integrated into the proposed algorithm. The flow chart of robot force control is shown in Figure 1. The actual force *f*_*e*_ is processed by a first-order low-pass filter to remove high-frequency noise. The difference between the actual force and the reference force is passed through the impedance controller to obtain the offset displacement of the robot. The offset displacement is compensated by integrating the DQN strategy and a compensation strategy based on the physical model of the skin. The compensations of the two different DQNs are *a*^1^ and *a*^2^, the compensation based on the physical model of the skin is *u*^*s*^, and the compensation after fusing offset displacement and strategy is *u*_*f*_. This compensation is sent to the internal displacement controller of the robot, thereby indirectly adjusting the contact state between the robot and the outside world.

## 3 Decision-making process of different strategies

### 3.1 Robot displacement compensation process with DQN strategies

Manually optimizing the compensation displacement selection is very tedious and time-consuming, whereas the reinforcement learning algorithm can independently identify the optimal control strategy. The reinforcement learning algorithm uses the Markov decision process as its theoretical framework. In the Markov decision process, the contact force state between the robot and the skin is denoted by *s*, the agent selects the robot action *a* according to the current contact state, and the robot executes action *a* to change the robot state. Simultaneously, the agent obtains an immediate reward *r* and then continues to choose the action according to the state at the next moment. The final trajectory τ obtained by the agent is τ = {*s*_0_, *a*_0_, *r*_0_, *s*_1_, *a*_1_, *r*_1_, *s*_2_, *a*_2_, *r*_2_, _…_, *s*_*t*_, *a*_*t*_, *r*_*t*_, _…_, *s*_T_, *a*_T_, *r*_T_}, where *r*_*t*_ is the instant reward at the *t-*th moment, tϵ[0, T]. The robot-skin interaction process is used to maintain the actual force within a certain range, so the instant reward can be set as the distance between the actual force and the reference force:


(4) $$\begin{array}{l} r_{t}=-k_{r}\text{*}|{f_{r}}^{t}-{f_{e}}^{t}| \end{array}$$

where *k*_*r*_ is the proportional factor. The contact state *s* can be set as the force error and the change of the force error, namely,


(5) $$\begin{array}{l} s_{t}=\left[e_{t},{ė}_{t}\right] \end{array}$$

where *e*_*t*_ is the force error at time *t*, $e_{t}={f_{r}}^{t}-{f_{e}}^{t}$, ė_*t*_ is the change of the force error, ė_*t*_ = *e*_*t*_ − *e*_*t*−1_. The robot action *a* is the impedance control compensation. Given a policy π, the discounted reward received by the trajectory τ of an interaction between the agent and the environment is:


(6) $$\begin{array}{l} R\left(\tau \right)=\overset{T-1}{\underset{t=0}{\sum }}\gamma^{t}r_{t+1}=\overset{T-1}{\underset{t=0}{\sum }}\gamma^{t}r\left(s_{t},a_{t},s_{t+1}\right) \end{array}$$

where γ is a discount factor between 0 and 1. When the time is *t*, the contact state is *s*_*t*_, and the action selection is *a*_*t*_, the expectation *E*(*R*_*t*_*|s*_*t*_, *a*_*t*_) of the defined discounted return *R* is the state-action value function, that is, the Q-function:


(7) $$\begin{array}{l} Q\left(s_{t},a_{t}\right)=E\left[R\left(t\right)|S_{t}=s|A_{t}=a\right] \end{array}$$

where *E* is the expectation and *S* and *A* are the sets of states and actions, respectively.

In the Q-learning algorithm, for each state *s*, the agent adopts the ε-greedy strategy. In the first action value function table, an action *a*_*t*_ is selected, and then the action *a*_*t*_is executed and transferred to the next state *s*_*t*_. In the second action value function table, an action *a*__*t*+_1_ that maximizes *Q*(*s*__*t*+_1_, *a*__*t*+_1_) is selected according to the state *s*__*t*+_1_, and the predicted value and target value are used to update the Q-value function. The prediction value uses the current state and the known Q-value function to estimate the Q-value of an action being taken in the current state, and the prediction value is *Q*(*s*_*t*_, *a*_*t*_). The target value updates the Q-value function, which is *r*_*t*_ + γmax*Q*(*s*__*t*+_1_, *a*__*t*+_1_), and the Q-value function gradually adjusts the Q-value through the difference between the predicted value and the target value:


(8) $$\begin{array}{l} Q\left(s_{t},a_{t}\right)\leftarrow Q\left(s_{t},a_{t}\right)+\alpha \left[r_{t}+\gamma \max Q\left(s_{t+1},a_{t+1}\right)-Q\left(s_{t},a_{t}\right)\right] \end{array}$$

where γ is the discount factor (0 ≤ γ ≤ 1) and α represents the learning rate of the model.

Through the learned Q-value function, the agent selects the action with the highest Q-value according to the current state to be the optimal strategy π^*^:


(9) $$\begin{array}{l} \pi *=\arg\max Q\left(s_{t},a_{t}\right) \end{array}$$

However, the state space of robot-skin contact is high-dimensional. To calculate the value function *Q*(*s, a*) in the state and action space, the neural network fitting method can be used to fit the action value function. However, if directly using one neural network updates the Q-learning algorithm, that is, the Q-value *r*_*t*_ + γmax*Q*(*s*__*t*+_1_, *a*__*t*+_1_) and target Q value *Q*(*s*_*t*_, *a*_*t*_) are the same network structure with the same parameters, the predicted value and the target value will change together, which increases the possibility of model oscillation and divergence to some extent. To address this, the predicted value deep neural network *Q(s, a*, θ) and the target value deep neural network *Q*(*s, a*, θ^−^) are used. When training parameters, samples are usually strongly correlated and non-static; if the data are applied directly, the model will have difficulty converging and the loss values will constantly fluctuate. The DQN algorithm introduces a mechanism for replaying experience: at each stage, the predicted value deep neural network executes action *a* through the ε-greedy strategy, namely:


(10) $$\begin{array}{l} a=\{\begin{array}{cc} \text{arg max }Q\left(s,a,\theta \right) & l-\varepsilon \\ \text{random action} & \varepsilon \end{array} \end{array}$$

After the experience sample data are obtained, the state and action data are stored in the experience pool. When the predictive value network needs to be trained, minibatch data are randomly selected from the experience pool for that training. On the one hand, introducing the experience pool replay mechanism makes backing up rewards easy; on the other hand, using a small number of random samples helps eliminate the correlation and dependence between samples. The loss function of the deep neural network for the predicted value is set to Mnih et al. (2015):


(11) $$\begin{array}{l} L\left(\theta \right)\;=\;E\left[{\left(y-Q\left(s,a,\theta \right)\right)}^{2}\right] \end{array}$$

where *L* represents the loss function and *y* is the value of the target network, as follows:


(12) $$\begin{array}{l} y_{t}=r_{t}+\gamma^{*}\max\left[\overset{⌢}{Q}\left(s_{t+1},\;a_{t+1},\theta^{-}\right)\right] \end{array}$$

In the initial state, the parameter θ of the predicted value network is the same as the parameter θ^−^ of the target network. Equation 11 is used to optimize the parameters of the predicted value network by gradient descent, and the parameter θ in the predicted network is updated. After the agent collects *G* group experience samples and *N* training iterations, the θ of the prediction network is copied to the θ^−^ of the target network, i.e., *Q^* = *Q*. As the above steps are repeated, the parameters of the predictor network are continuously updated to improve the predictive power and performance of the network, whereas the parameters of the target value network are relatively stable and are only periodically copied from the predictor network. The fitting ability of the Q-value function is gradually optimized, and the agent selects the action with the highest Q-value as the current optimal decision according to the current state.

The neural network is constructed by a multilayer feedforward neural network, which consists of an input layer, multiple hidden layers, and an output layer. The contact state of the robot is passed through the input layer to the output layer along with connections between neurons in the hidden layer. Finally, the Q-value is output. In the hidden layer, the neural network first calculates the net activation value *Z^l^* of the neurons in the *l*-th layer according to the activation value *U*^l − 1^ of neurons in layer (*l*-1)-th and then uses an activation function to obtain the activation value of neurons in the *l*-th layer. Let the input be the state value of the robot, that is, *U*^0^=*s*; information is disseminated by continuously iterating the following equation (Li et al., 2012):


(13) $$\begin{array}{l} Z^{l}\;=\;W^{l}U^{l-1}+B^{l}\\ U^{l}\;=\;\varphi \left(Z^{l}\right) \end{array}$$

where *W*^*l*^ is the weight of the *l-*th layer; *b*^*l*^ is the bias of the *l-*th layer; *Z*^*l*^ is the net activation value of the *l-*th layer; *U*^*l*^ is the activation value of the *l-*th layer; and φ is the activation function. The ReLU activation function is selected:


(14) ReLU(Z)={ZZ≥00Z<0

The parameters of the neural network are trained by backpropagation, and the partial derivative of the loss function for each parameter in the network is calculated. Then, the chain rule is used to backpropagate these partial derivatives to each layer in the network, thereby updating the parameters to minimize the loss function. The error term δ^*l*^ for the *l*-th layer is calculated by backpropagation, and the sensitivity of the final loss to the neurons in layer *l* is defined as Shi (2021):


(15) $$\begin{array}{l} \delta^{l}\;≜\frac{\partial L\left(\theta \right)}{\partial Z^{l}} \end{array}$$

The derivative of each layer parameter is:


(16) $$\begin{array}{l} \begin{array}{c} \frac{\partial L\left(\theta \right)}{\partial W^{l}}=\delta^{l}{\left(U^{l-1}\right)}^{T}\\ \frac{\partial L\left(\theta \right)}{\partial b^{l}}=\delta^{l} \end{array} \end{array}$$

where δ^*l*^ is the error term of neurons in the *l* layer. Finally, the neural network parameters are updated:


(17) $$\begin{array}{l} W^{l}\leftarrow W^{l}-\alpha \delta^{l}{\left(U^{l-1}\right)}^{T}+\lambda W^{l}\\ b^{l}\leftarrow b^{l}-\alpha \delta^{l} \end{array}$$

where α is the learning rate and λ is the regularization coefficient.

### 3.2 Dynamics models of reinforcement learning

The agent of reinforcement learning must go through trial and error when improving the policy and conducting multiple experiments in the actual interaction to achieve the desired result. However, frequent trial and error processes will not only negatively impact the interactive experience but also cause damage and pain to the human skin due to repeated friction. Therefore, fast convergence of the algorithm during robot-skin contact is crucial. Since the DQN algorithm is a model-free algorithm, it must conduct multiple experiments to obtain sufficient data. To accelerate the convergence, dynamic models of the reinforcement learning environment can be constructed so that DQN can iteratively train in a virtual environment, reducing the number of actual training and improving the practicality of the algorithm.

#### 3.2.1 BP neural network dynamics model

Since skin has biological characteristics, the mechanical characteristics of skin are non-linear. The dynamic model of the robot is also non-linear, so the contact process between the two can be set as a non-linear system; the BP neural network has non-linear mapping capabilities, so it can construct the relationship between the contact state and robot displacement. The network inputs the contact state *e*_*t*_, ė_*t*_, and the compensation displacement *a* of the robot, and the output state is *e*__*t*+_1_, ė_*t*+1_. The dynamics model constructed by the BP neural network is composed of the data of multiple impedance algorithms, and the fitted model is as follows:


(18) $$\begin{array}{l} s_{t+1}=\varphi \left(s_{t},a_{t},NeT1.W,NeT1.b\right) \end{array}$$

where *NeT1.W* and *NeT1.b* are the weight and bias parameters in the BP neural network. The network can be updated through Equations 13–17. After the BP neural network constructs the environmental dynamics model, the DQN algorithm can be used to train the strategy offline in this model. Once the compensation strategy satisfies Equation 9, the output compensation displacement *a*^1^ can be obtained.

#### 3.2.2 LSTM neural network dynamics model

The presence of noise information in the robot state data is likely to lead to inaccurate information in the network results. A recurrent neural network can establish the correlation of state model information in time series and integrate multiple state information according to the characteristics of spatiotemporal context information; through doing so, the network can reduce noise interference and purify the sample set so that a more accurate state model can be obtained. A certain connection exists between the robot state data; the long short-term memory (LSTM) neural network has short-term memory ability, so it can build further connections between the data. Neurons in LSTM can receive information not only from other neurons but also from themselves, forming a network structure with loops. The LSTM better aligns with the structure of the biological neural network than with the feedforward neural network, and the fitted model is as follows:


(19) $$\begin{array}{l} S_{t+1}=\phi \left(S_{t},A_{t},NeT2.W,NeT2.b\right) \end{array}$$

LSTM can effectively capture and store long-term dependencies by introducing memory units and gating mechanisms. The gating mechanism controls the path of information transmission; the forget gate *f*_*t*_ determines whether to retain the memory unit *C*__*t*_−1_ at the previous moment, and the input gate controls how much information must be saved at the current moment. The output gate *o*_*t*_ controls how much information the memory state *C*__*t*_−1_ at the current moment must output to the hidden state *H*_t_. The memory unit in LSTM is a linear structure that can maintain the chronological flow of information. When *f*_*t*_ = 0 and *i*_*t*_ = 1, the memory unit clears the historical information; when *f*_*t*_ = 1 and *i*_*t*_ = 0, the memory unit copies the content of the previous moment, and no new information is written. The key operations of LSTM are expressed as follows (Shi et al., 2015):


(20) $$\begin{array}{l} i_{t}=\sigma (NeT2.W_{xi}X_{t}+NeT2.W_{hi}H_{t-1}\\ +NeT2.W_{ci}⊙C_{t-1}+NeT2.b_{i})\\ f_{t}=\sigma (NeT2.W_{xf}X_{t}+NeT2.W_{hf}H_{t-1}\\ +NeT2.W_{cf}⊙C_{t-1}+NeT2.b_{f})\\ o_{t}=\sigma (NeT2.W_{xo}X_{t}+NeT2.W_{ho}H_{t-1}\\ +NeT2.W_{co}⊙C_{t-1}+NeT2.b_{0})\\ C_{t}=f_{t}⊙C_{t-1}+i_{t}⊙\text{tanh}(NeT2.W_{xc}X_{t}\\ +NeT2.W_{hc}H_{t-1}+NeT2.b_{c})\\ H_{t}=o_{t}⊙\text{tanh}(C_{t}) \end{array}$$

where, *i*_t_, *f*_t_, and *o*_t_ represent the input gate, forget gate, and output gate in the LSTM, respectively; *t* represents the period, *X*_*t*_ denotes the input at the current moment, *C*_*t*_ represents the memory state, *H*_*t*_represents the hidden state, and *Net*2.*W*_*f*_, *Net*2.*W*_*i*_, *Net*2.*W*_*c*_, and *Net*2.*W*_*o*_ are the weights of the forget gate, input gate, estimated state, and output gate, respectively. ⊙ denotes the Hadamard product. σ is a logistic function with an output interval of (0,1), and *H*__*t*_−1_ is the external state at the previous moment.

After the LSTM neural network constructs the dynamics model, the DQN algorithm can also be used to train offline in the constructed model, and the output compensation displacement *a*^2^ can be obtained.

### 3.3 Robot displacement compensation strategy with a skin mechanics model

For the skin contact environment, the amount of skin extrusion deformation first increases and then slowly increases as pressure increases, which has the non-linear elastic characteristics of compliant materials. The Hunt-Crossley skin mechanics model defines the relationship between the force on the skin and the depth of extrusion as a power function, which can conform to the non-linear elastic and viscous mechanical properties of skin-like soft material objects. In the one-dimensional direction, when the skin is squeezed, the deformation force of the skin is Schindeler and Hashtrudi-Zaad (2018):


(21) $$\begin{array}{l} f_{s}=k_{s}{\left(\left|x-x_{e}\right|\right)}^{\beta }+b_{s}{\left(\left|ẋ-{ẋ}_{e}\right|\right)}^{\beta } \end{array}$$

where *f*_*s*_ is the force generated by skin deformation; *x* is the coordinate of the robot when it is deformed; *x*_*e*_ are the initial coordinates of the skin when it is not deformed by force; |*x*-*x*_e_| is the amount of deformation; *k*_*s*_ and *b*_*s*_ are the elasticity and damping coefficients, respectively; and *b*_*s*_is the power exponent, determined by the nature of the skin in the local contact area. The parameters of the skin of different parts of the human body differ in certain ways, and the parameters in Equation 21 also change, so directly using Equation 21 to calculate the parameters online is cumbersome. Therefore, when the robot moves along the skin, the axis is fine-tuned in the Z-axis direction, that is, ẋ≈0; for calculation ease, Equation 21 is simplified to:


(22) $$\begin{array}{l} f_{s}=k_{s}{\left(\left|x-x_{e}\right|\right)}^{\beta } \end{array}$$

The parameters *k*_*s*_ and β are fitted by an offline collection of deformation and contact force data of different parts of the body by using the least square method. Therefore, the online compensation displacement of the robot is:


(23) $$\begin{array}{l} u^{s}=\sqrt[{\beta }]{\frac{f_{e}}{k_{s}}}-\sqrt[{\beta }]{\frac{f_{r}}{k_{s}}} \end{array}$$

where *u*^*s*^ is the compensation displacement based on displacement compensation with skin mechanics.

## 4 Force control strategy fusion process based on the GMM/GMR algorithm

All strategies for the environment dynamics model built by the BP neural network or the LSTM neural network are offline training strategies, and some errors will still exist in the actual process regardless of which strategy is chosen. Although the robot displacement compensation strategy under the physical model of skin mechanics is an online strategy, experience data cannot improve it. Therefore, the fusion strategy is employed to effectively fuse the prediction results of different data sources or models to improve the accuracy and robustness of the overall prediction.

The GMM/GMR algorithm is flexible, highly efficient, adaptable to multivariate data, interpretable and robust. These advantages can support the fusion of robot force control strategies. GMM is a probability model based on a Gaussian distribution that assumes the data are a mixture of several Gaussian distributions. By training the data, the GMM can learn the parameters (mean and covariance matrix), as well as the weight, of each Gaussian distribution. These parameters can be used to describe the data distribution and to generate new samples.

Under the three strategies, the robot may obtain three different predicted robot force trajectories, that is, $\left\{{\left\{t_{n},a_{n}^{1}\right\}}_{n=1}^{N_{m}},{\left\{t_{n},a_{n}^{2}\right\}}_{n=1}^{N_{m}},{\left\{t_{n},{u^{s}}_{n}\right\}}_{n=1}^{N_{m}}\right\}$, and the predicted values of the deep neural network model and the skin mechanics model. Here, *n* is the number of samples, *N*_*m*_ is the length of the trajectory, *t* is the time information, *a*^1^, *a*^2^, and *u*^*s*^ are the output compensation displacements of the robot, *u* represents the three kinds of compensation displacements, and the GMM can model the joint probability distribution *P*(*t, u*) of the input and output variables in the sample as follows (Man et al., 2021):


(24) $$\begin{array}{l} p\left(t,u\right)\sim \overset{M}{\underset{m=1}{\sum }}\pi_{m}N\left(\mu_{m},\Sigma_{m}\right) \end{array}$$

where *M* is the number of Gaussian components in the GMM. π_*m*_, μ_*m*_, and Σ_*m*_ represent the prior probability, mean and covariance of the *m*-th Gaussian component, respectively, and μ_*m*_ and Σ_*m*_ are defined as follows:


(25) $$\begin{array}{l} \mu_{m}=\left[\begin{array}{c} \mu_{t,m}\\ \mu_{u,m} \end{array}\right]\\ \Sigma_{m}=\left[\begin{array}{cc} \Sigma_{tt,m} & \Sigma_{tu,m}\\ \Sigma_{ut,m} & \Sigma_{uu,m} \end{array}\right] \end{array}$$

The parameters of the GMM are iteratively optimized through the expectation-maximization (EM) algorithm (Hu et al., 2023), the posterior probability of each sample point belonging to each Gaussian component is calculated, and the mean value, covariance matrix and mixing coefficient of the Gaussian component are updated. After obtaining the trained GMM model, GMR is used to make a regression prediction on the robot force trajectory. The posterior probability of each Gaussian component is first calculated, and the weighted sum of the posterior probability is used to obtain the weighted Gaussian component mean and covariance matrix. A new trajectory point is then obtained by sampling from each Gaussian component. GMR is used to predict the conditional probability distribution of the corresponding trajectory of a new input:


(26) $$\begin{array}{l} p(u^{*}|t^{*})=\sum_{m=1}^{M}h_{m}(t^{*})N({\bar{\mu }}_{m}(t^{*},{\bar{\Sigma }}_{m}) \end{array}$$

where t^*^ and u^*^ are the predicted time and compensation displacement, respectively, and *h*_m_, ${\bar{\mu }}_{c}$, and ${\bar{\Sigma }}_{m}$ are calculated as follows:


(27) $$\begin{array}{l} h_{m}(t^{*})=\frac{\pi_{m}N(t^{*}|\mu_{t,m},\Sigma_{tt,m})}{\sum_{i=1}^{M}\pi_{i}N(t^{*}|\mu_{t,i},\Sigma_{tt,i})}\\ {\bar{\mu }}_{c}(t^{*})=\mu_{u,m}+\Sigma_{tt,m}\Sigma_{tt,m}^{-1}(t^{*}-\mu_{t,m})\\ {\bar{\Sigma }}_{m}=\Sigma_{uu,m}-\Sigma_{ut,m}\Sigma_{tt,m}^{-1}\Sigma_{tu,m} \end{array}$$

For calculation convenience, Equation 26 can be approximated as


(28) $$\begin{array}{l} p\left(u^{*}|t^{*}\right)\approx N\left(\hat{\mu }\hat{\Sigma }\right) \end{array}$$

where $\hat{\mu }=\overset{M}{\underset{m=1}{\sum }}h_{m}\left(t^{*}\right){\bar{\mu }}_{c}\left(t^{*}\right)$, $\hat{\Sigma }=\overset{M}{\underset{m=1}{\sum }}h_{c}\left(t^{*}\right){{\bar{\mu }}_{m}}^{T}\left(t^{*}\right)+{\bar{\Sigma }}_{m}-\hat{\mu }{\hat{\mu }}^{T}$, the central distribution of u* is obtained according to the probability distribution in *p*(u^*^|t^*^), and *u*_*f*_ is the final fusion strategy.

## 5 Experimental setup of the force control based on the GMM/GMR algorithm

**Figure 2:**
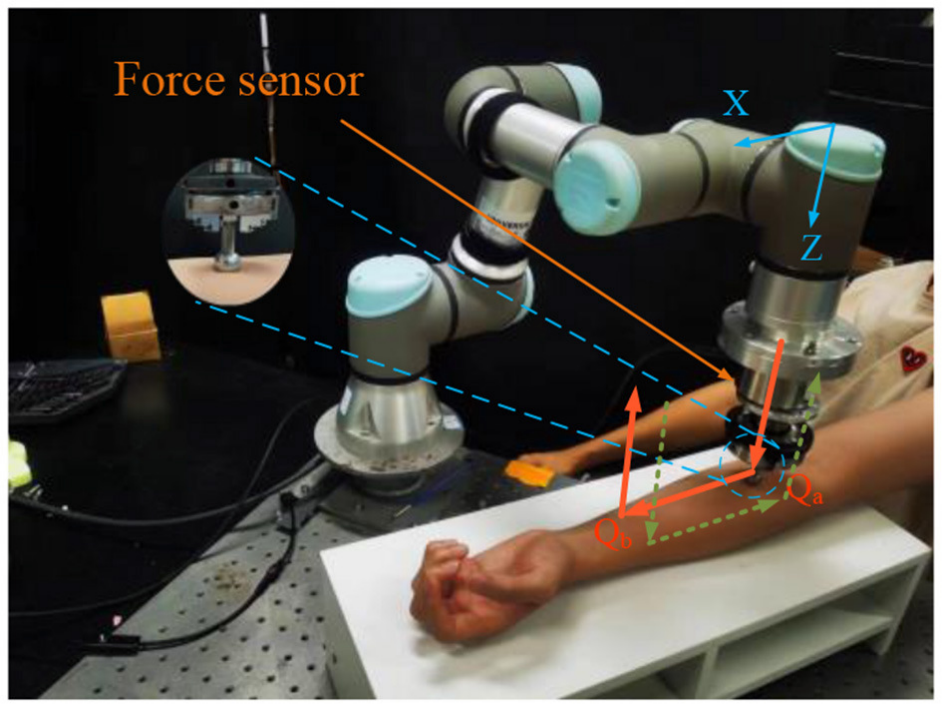
Schematic diagram of the robot tracking process along the skin.

A schematic diagram of the experiment is shown in Figure 2. In this experiment, the robot squeezes the skin vertically along the Z direction at a speed of 2 mm/s. When the robot reaches the reference force *f*_*r*_ along the Z direction, i.e., point Q_a_ in the figure, the robot stops moving in the Z direction, enters force control mode to move horizontally along the X direction at a speed of 2 mm/s for 5 s until reaching point Q_b_, the robot then leaves the human skin vertically. The second trajectory is in the opposite direction, starting from Q_b_ to Q_a_. The force sensor is an ME-FKD40, and the force signal is collected by a backoff module and transmitted to the robot controller, the control system works at a frequency of 50 Hz, and the robot force control only tested while moving from point Q_a_ to Q_b_ or from point Q_b_ to Q_a._

**Figure 3:**
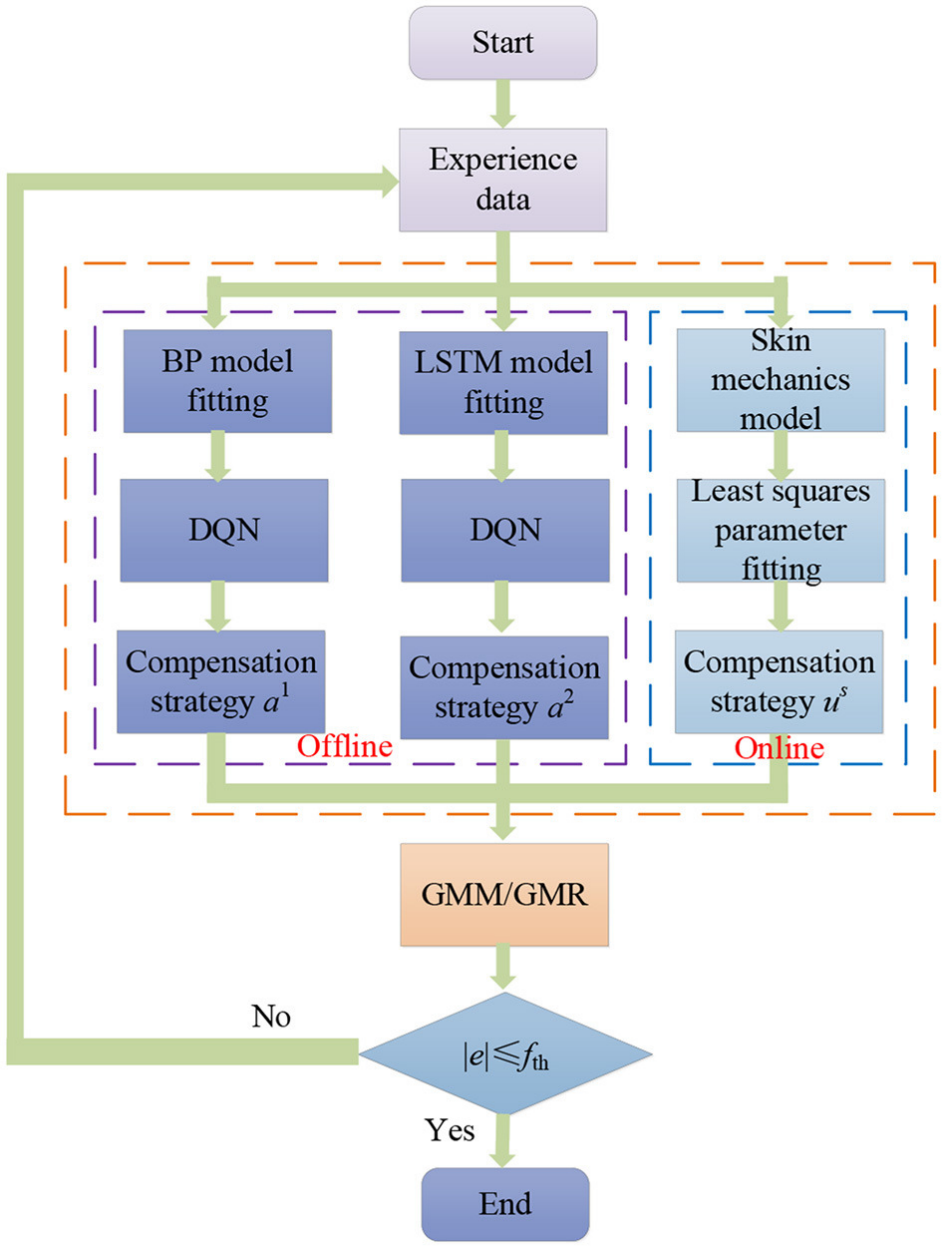
Experimental flowchart.

The force control based on the GMM/GMR algorithm experimental process is shown in Figure 3. Multiple sets of impedance data parameters are used to obtain the robot contact states and displacements in the Z-direction to get experience data. When different impedance strategies are implemented, the difference between the force on the end of the robot and the reference force *e*_*t*_, the rate of change of the error ė_*t*_ and the offset displacement Δ*x*_*t*_ of the robot are collected, which can be used for fitting the BP and LSTM neural network model. The least squares algorithm is used to fit parameters in the skin mechanics model. The DQN strategy is obtained through offline training, and the compensation strategy based on the skin mechanics model is obtained through online calculation. If the force error obtained by the force control based on the GMM/GMR algorithm is greater than the expected threshold ±0.2 N, the obtained data can be added to the database. Then, the BP neural network can be updated again, and experiments can be iterated until the error between the force in the Z-direction and the reference force is within the set range, namely, ±0.2 N.

## 6 Robot-skin contact experiment results and analysis

**Figure 4:**
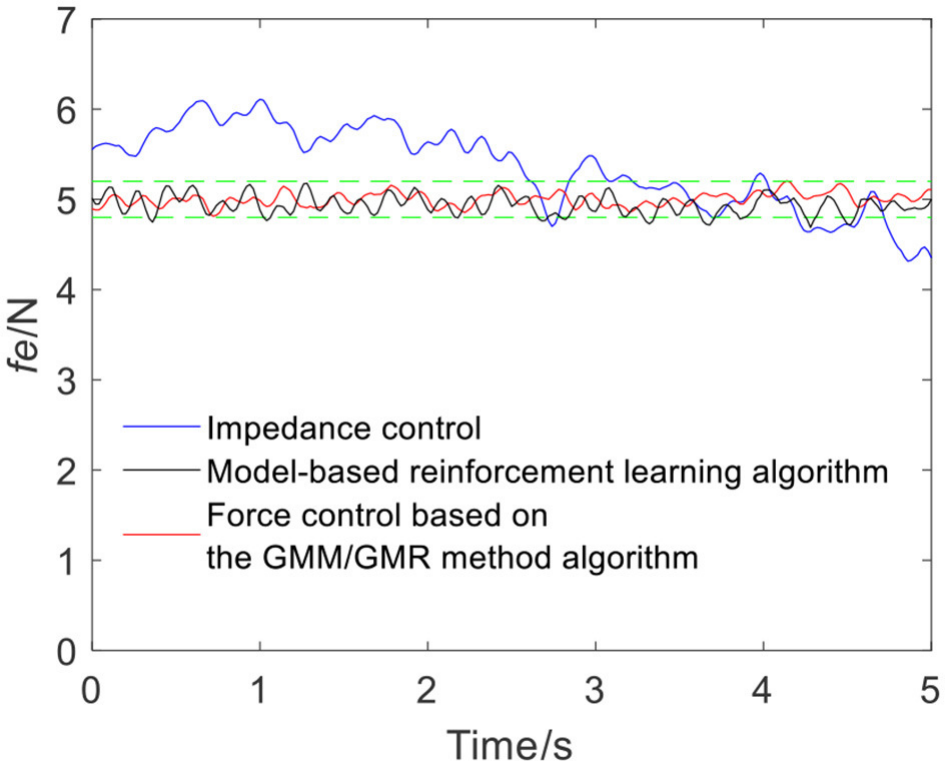
Tracking results comparison of impedance control and the force control based on the GMM/GMR algorithm (volunteer A, Q_a_ to Q_b_).

To ensure the volunteers' safety, when the robot applies force on the skin surface, a gentle force application strategy is adopted, and the reference force of the robot is set to 5 N, i.e., *f*_*r*_ = 5 N. In the impedance control strategy, the parameters are manually adjusted to *m*_*d*_ = 10, *b*_*d*_ = 6, and *k*_*d*_ = 700 according to experience. When the robot moves along the skin from point Q_a_ to Q_b_, the tracking force obtained by impedance control is illustrated by the blue line in Figure 4. It can be seen from the force signal that the robot maintains contact with volunteer A, meanwhile, the force exhibits certain fluctuations. The comparison between impedance control and the force control based on the GMM/GMR algorithm fusing different compensation strategies is shown in Figure 4. The force control based on the GMM/GMR algorithm is significantly smoother than impedance control, and the control effect is significantly improved.

**Figure 5:**
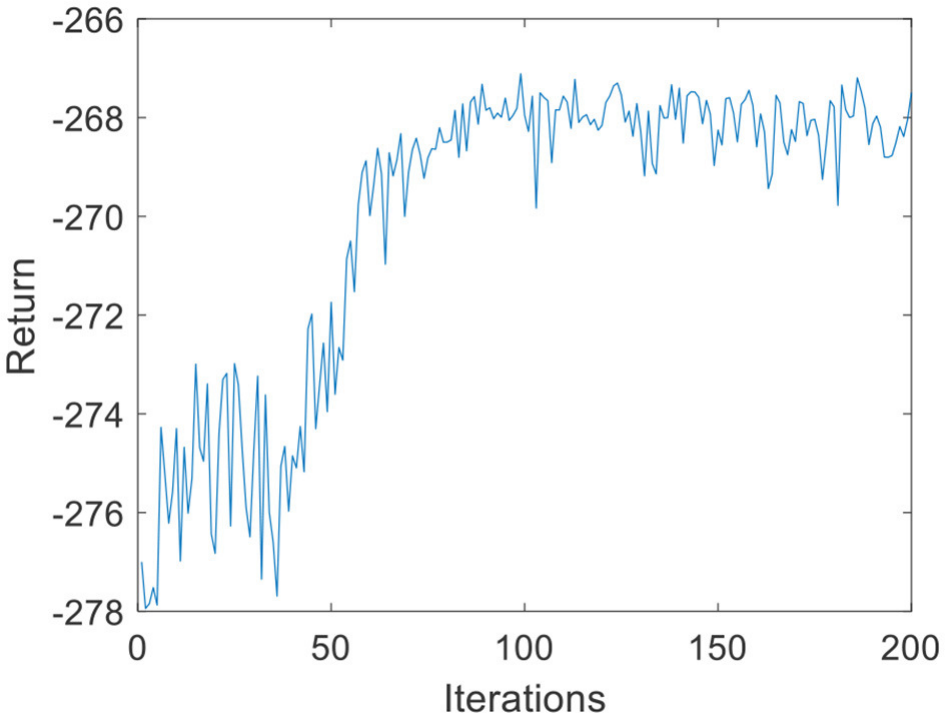
DQN training process under the BP neural network (volunteer A).

**Figure 6:**
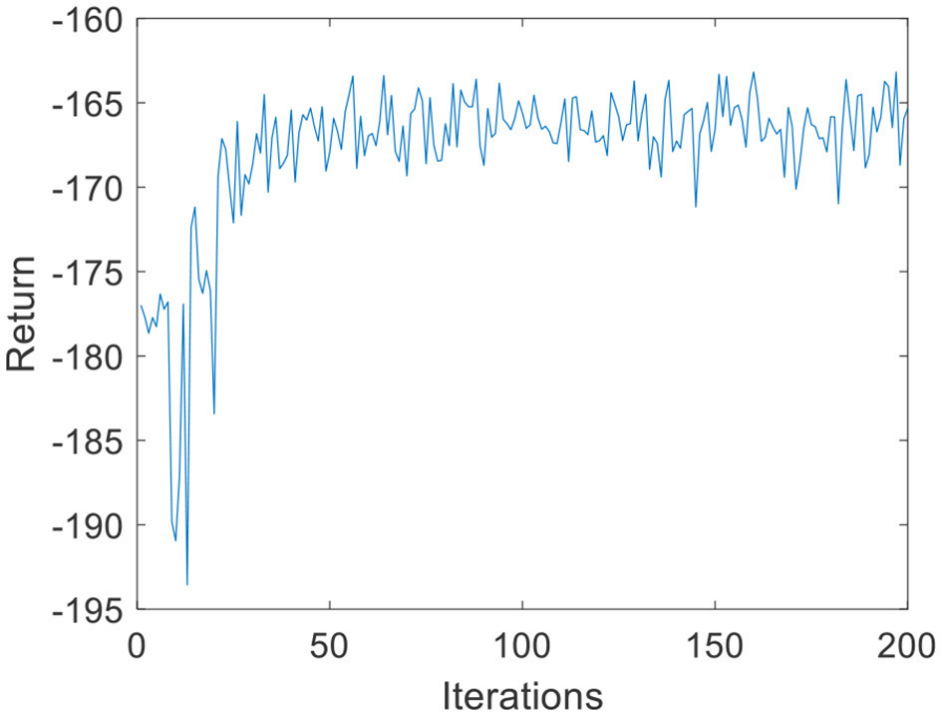
DQN training process under the LSTM neural network (volunteer A).

Due to the small amount of input and output data, in the environmental dynamics model constructed by the BP neural network, the range of action *a* is [0:0.01:0.2] with a total of 20 actions. When the force error is negative, *a* chooses the opposite direction, which can reduce invalid searches. The output is the state at the next moment. The middle node of the neural network is set to 30, and the number of layers of the neural network is set to 2. In the LSTM neural network, the intermediate nodes of the neural network are set to 20. For the input data *s* of the DQN, the Q-values, which are 1-dimensional data, are the output. Due to the parameter dimensions and the small amount of data, the deep neural network is much smaller than the image dimension; therefore, the number of layers of the neural network is set to 2, and the number of nodes in each layer is set to 30. The step size of the DQN is set to T = 200, *G* = 200, *k*_*r*_ = 10 in Equation 3, ε is 0.1 in Equation 11, and the total number of iterations *N* is 200. The iterative process of the DQN algorithm under the environmental dynamics model of the BP neural network is shown in Figure 5. As the number of iteration data increases, the algorithm converges after ~50 iterations. The iterative process of the DQN algorithm under the environmental dynamics model of the LSTM neural network is shown in Figure 6. As the number of iteration data increases, the algorithm converges after ~40 iterations. In the online strategy based on the skin mechanics model, the force data of different skins are chosen to fit the parameters of the skin mechanics model, namely, *k*_*s*_ = 0.015 and β = 2.5 in Equation 24. In the GMM/GMR algorithm, *M* is equal to 2, and the length of the trajectory *N*_*m*_ = 20.

**Figure 7:**
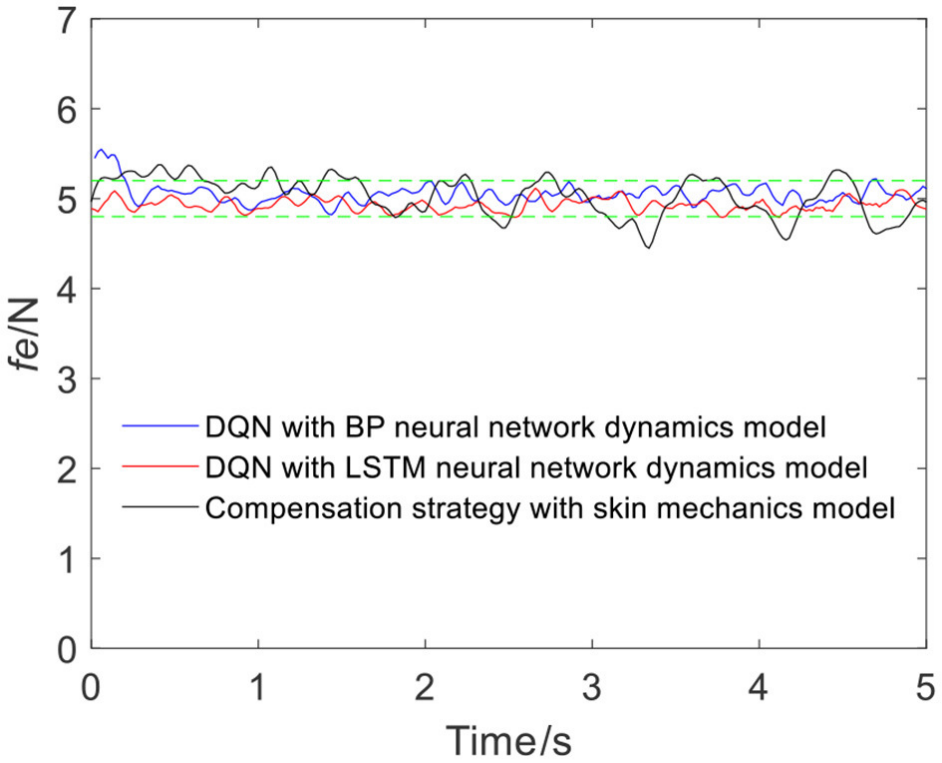
The experimental results of three different strategies (volunteer A, Q_a_ to Q_b_).

**Figure 8:**
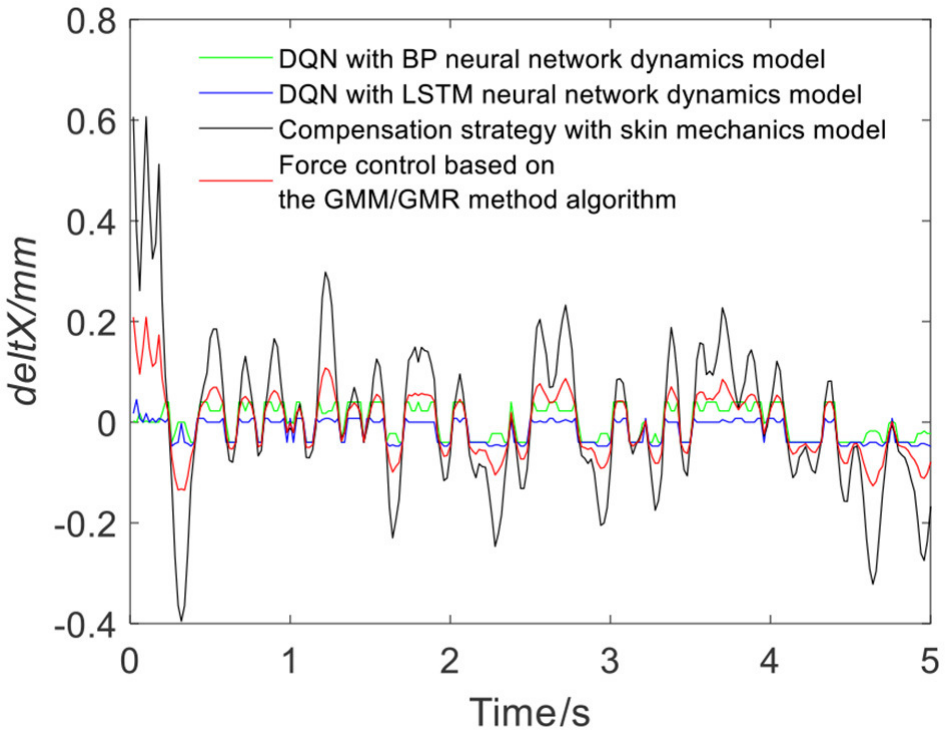
Robot offset displacement of different strategies (volunteer A, Q_a_ to Q_b_).

Figure 7 shows the results of three different force control strategies that are run separately. All three algorithms achieve good results, but they exhibit relatively large fluctuations. Figure 8 depicts the offset displacement strategies of three different strategies under the robot force control based on the GMM/GMR algorithm. The DQN with the BP neural network dynamics model and the LSTM dynamics model are relatively conservative, while the algorithm based on the skin mechanics model is relatively radical.

**Figure 9:**
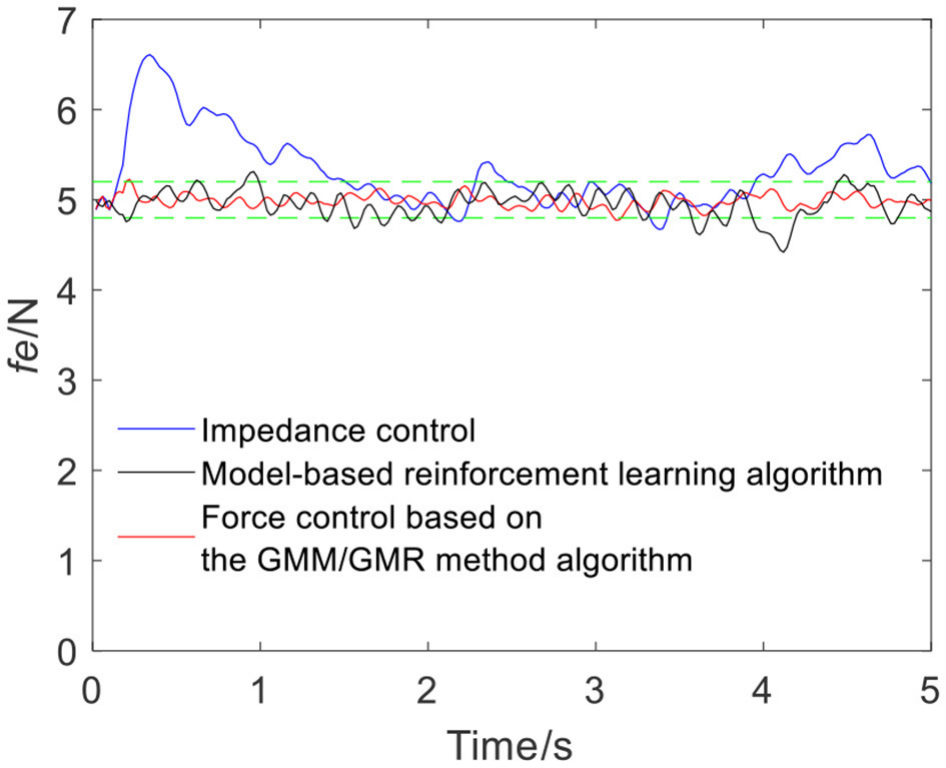
Tracking results comparison of impedance control and the robot force control based on the GMM/GMR algorithm (volunteer B, Q_a_ to Q_b_).

**Figure 10:**
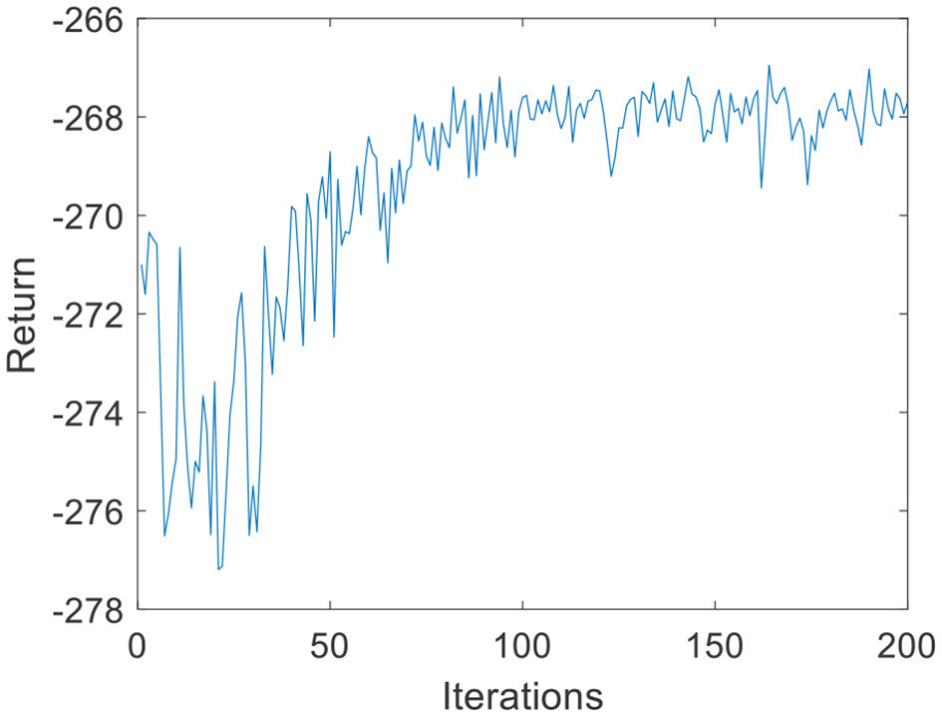
DQN training process under the BP neural network (volunteer B).

**Figure 11:**
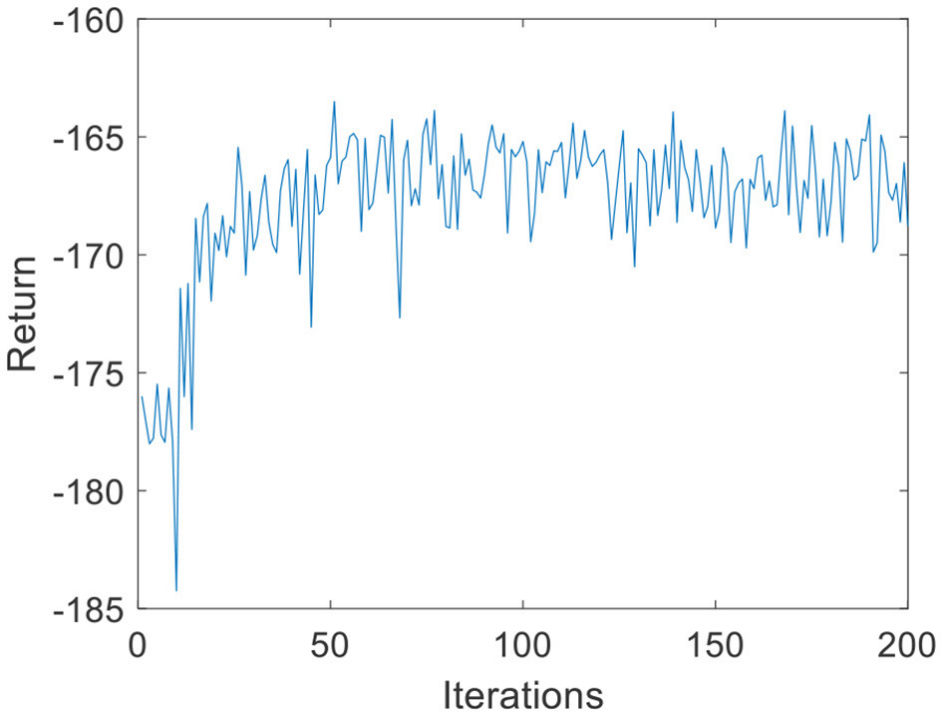
DQN training process under the BP neural network (volunteer B).

**Figure 12:**
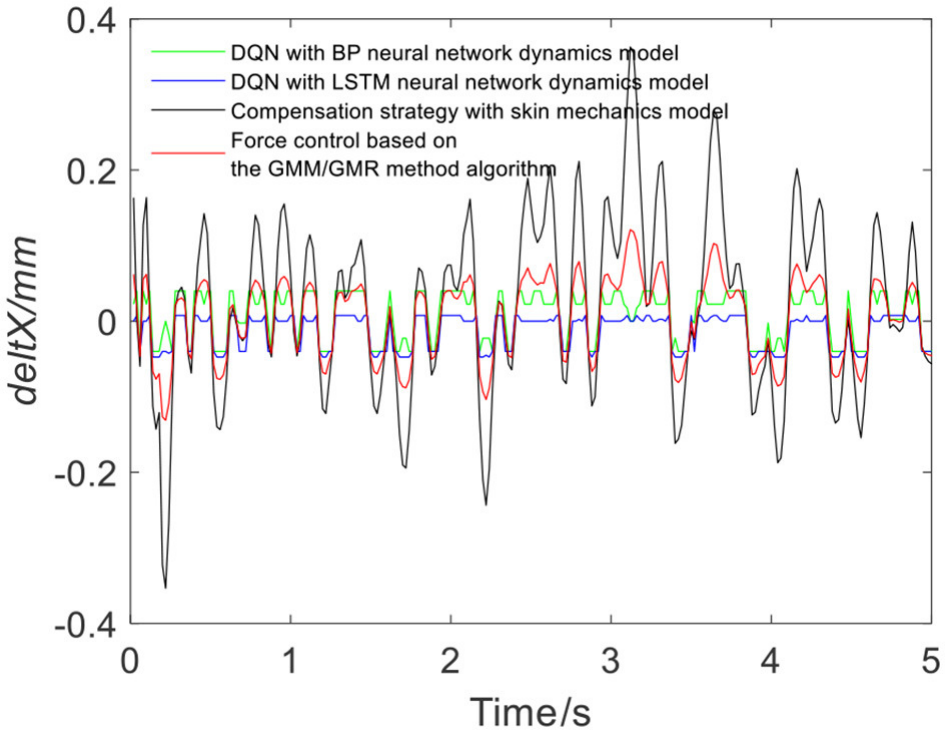
Robot offset displacement of different strategies (volunteer B, Q_a_ to Q_b_).

**Figure 13:**
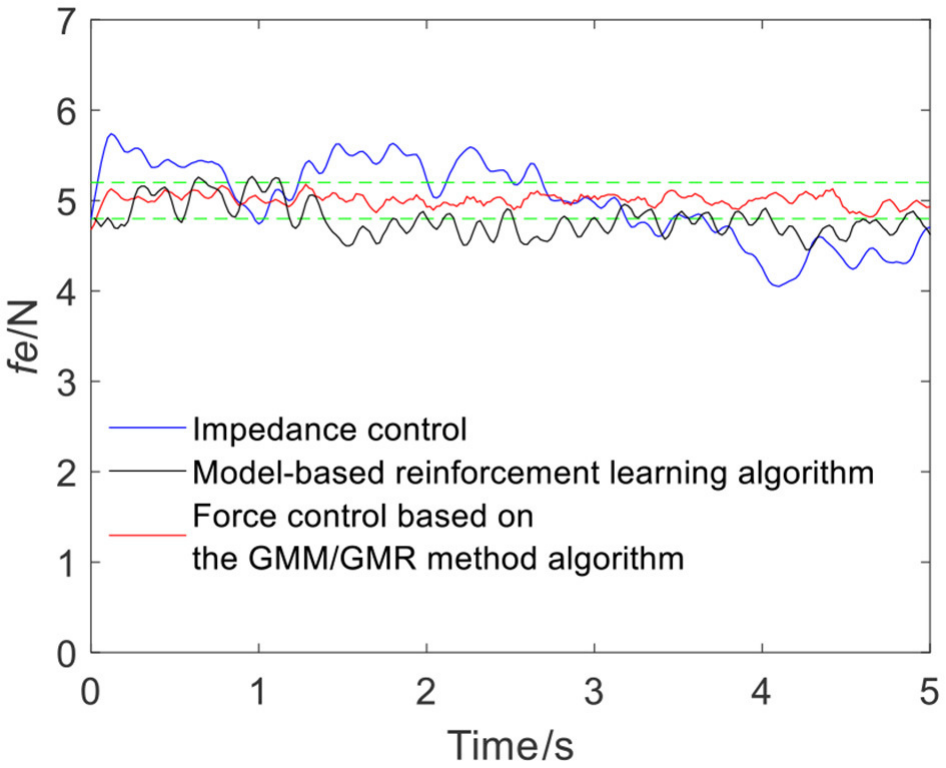
Tracking results comparison of impedance control and the force control based on the GMM/GMR algorithm (volunteer A, Q_b_ to Q_a_).

**Figure 14:**
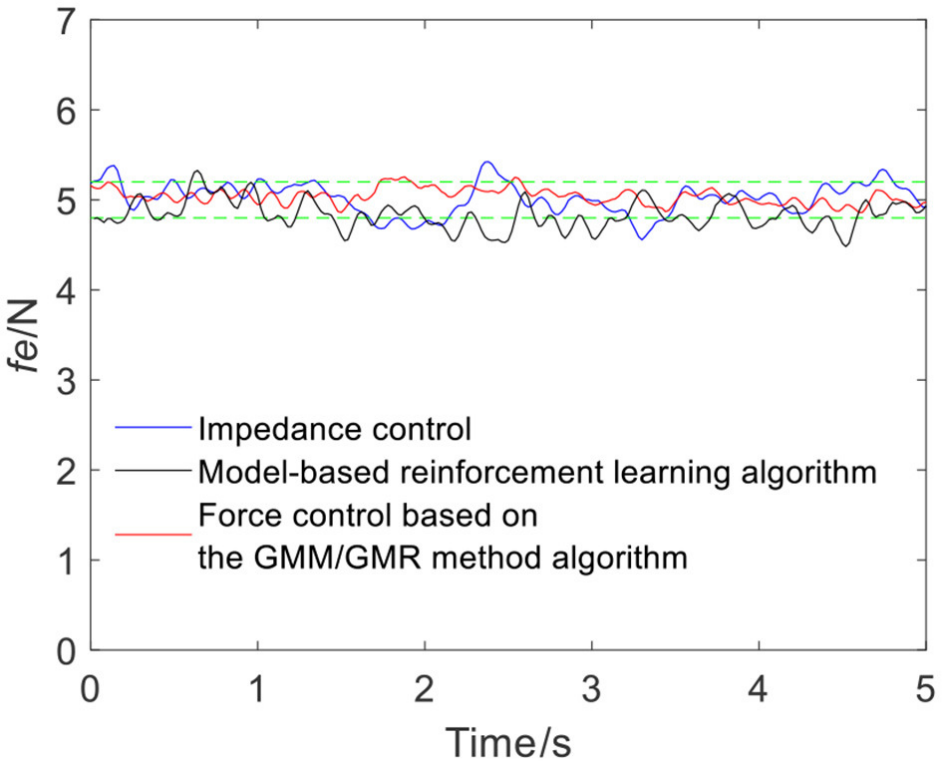
Tracking results comparison of impedance control and the force control based on the GMM/GMR algorithm (volunteer B, Q_b_ to Q_a_).

To verify the versatility of the proposed algorithm, the arms of different volunteers are tracked with the robot force control based on the GMM/GMR algorithm. The parameters are consistent with the first experiment. The comparison results of the impedance control process and robot force control based on the GMM/GMR algorithm are shown in Figure 9. Similar to the effect of volunteer A, the obtained force signal also fluctuates to a certain extent with impedance control. The robot force control based on the GMM/GMR algorithm's force signal is significantly smoother than that of the impedance control strategy, the error against the reference force is stable within a certain range, and the control effect is significantly improved. The return values of reinforcement learning with the BP neural network and the LSTM neural network dynamics model are shown in Figures 10, 11. Both gradually converge after ~50 iterations. The robot offset displacement of different strategies can be computed online as shown in Figure 12. The experiment of robot trajectory Q_b_ to Q_a_ is shown in Figures 13, 14, although the contact force signal obtained with impedance control under different external conditions has good contact effect, the force signal of force control strategy fusion algorithm is relatively smoother.

The intelligent algorithm for comparison is a model-based reinforcement learning algorithm, which is constructed by combining a neural network and a cross-entropy method for control parameter search. The obtained force is shown as the black line in Figures 4, 9, 13, 14. Compared with the impedance control algorithm in the four groups of experiments, the model-based reinforcement learning algorithm has better results. However, the force signal of the model-based reinforcement learning algorithm exceeds the threshold in some trajectories, such as in the second half of the force tracking on volunteer B in Figure 9, and the robot force control based on the GMM/GMR algorithm is more stable and has better versatility.

The error comparison between the impedance control, model-based reinforcement learning algorithm and robot force control based on the GMM/GMR algorithm is shown in Table 2. The error of force tracking with the robot force control based on the GMM/GMR algorithm includes the maximum absolute value |*e*|_*max*_, the mean absolute error |ē| and the standard deviation of error σ_*e*_. In the robot force control experiment of the trajectory from Q_a_ to Q_b_ on different volunteers, the mean absolute errors |ē| of the robot force control based on the GMM/GMR algorithm were significantly reduced by 87.5 and 80%, respectively, compared with that of the impedance control strategy. In the robot force control experiment of the trajectory from Q_b_ to Q_a_ on different volunteers, the mean absolute errors |ē| of the robot force control based on the GMM/GMR algorithm were reduced by 85.7 and 45.7%, respectively. And all three types of errors had been significantly reduced, too. Compared with model-based reinforcement learning, the mean absolute errors |ē| of the robot force control based on the GMM/GMR algorithm were reduced by 35.7, 65.7, 74.4, and 60%, respectively.

**Table 2:** Error comparison of force control algorithms between impedance control, model-based reinforcement learning algorithm and the force control based on the GMM/GMR algorithm.

**Algorithm**	**|*e*|*_*max*_*/N**	**|ē|/N**	***σ_*e*_*/N**
Impedance control (volunteer A, Q_a_ to Q_b_)	1.1	0.49	0.46
Model-based reinforcement learning algorithm (volunteer A, Q_a_ to Q_b_)	0.31	0.095	0.1
Robot force control based on the GMM/GMR algorithm (volunteer A, Q_a_ to Q_b_)	0.21	0.061	0.074
Impedance control (volunteer A, Q_b_ to Q_a_)	0.95	0.38	0.45
Model-based reinforcement learning algorithm (volunteer A, Q_b_ to Q_a_)	0.58	0.13	0.16
Robot force control based on the GMM\GMR algorithm (volunteer A, Q_b_ to Q_a_)	0.26	0.054	0.068
Impedance control (volunteer B, Q_a_ to Q_b_)	1.6	0.33	0.39
Model-based reinforcement learning algorithm (volunteer B, Q_a_ to Q_b_)	0.54	0.25	0.18
Robot force control based on the GMM/GMR algorithm (volunteer B, Q_a_ to Q_b_)	0.37	0.064	0.087
Impedance control (volunteer B, Q_b_ to Q_a_)	0.44	0.14	0.17
Model-based reinforcement learning algorithm (volunteer B, Q_b_ to Q_a_)	0.51	0.19	0.15
Robot force control based on the GMM/GMR algorithm (volunteer B, Q_b_ to Q_a_)	0.25	0.076	0.087

The reason why the robot force control based on the GMM/GMR algorithm is better than the traditional impedance control is that the impedance control adjustment range is small. Although impedance control can ensure that the robot and skin remain in contact facing volunteers A and B, a fixed impedance parameter cannot ensure the accuracy of the robot-skin contact process. The accuracy of the model-based reinforcement learning strategy depends on whether the model conforms to reality. When the robot contact state exceeds the range of the model, there will be an error between the offline reinforcement learning strategy and the actual demand. However, when the robot force control based on the GMM/GMR algorithm faces unknown skin environments, the skin mechanics model can propose compensation strategies online and modify the robot state in real time, at the same time, the DQN with the BP and LSTM neural network models can provide the historical experience of offline learning. When the GMM/GMR algorithm integrates the two, the robot can obtain the advantages of both. The fusion strategy for volunteers A and B is relatively stable and has relatively good versatility.

## 7 Conclusions and future work

A robot force controller based on the GMM/GMR algorithm is proposed that combines different compensation strategies and is applied to robot-skin contact scenarios. The initial robot force control strategy is established by impedance control, the reinforcement learning algorithm and traditional control strategy are fused to compensate for the impedance control. Two environmental dynamics models of reinforcement learning are constructed to simulate the contact process between the robot and the skin, and accelerate the offline convergence of the reinforcement learning algorithm. The GMM/GMR algorithm fuses online and offline compensation strategies to improve the robustness and versatility of the algorithm and to adapt to different skin environments.

The experimental results show that the robot force control based on the GMM/GMR algorithm has good versatility and accuracy. Under 100 offline iterations, the reinforcement learning algorithm can select effective control parameters. The force can quickly converge to the reference force, and its error is stable within the range of ±0.2 N. The method has also achieved good results with different volunteers. Furthermore, for the force obtained by using the reinforcement learning algorithm, the maximum absolute value, the mean absolute error and the standard deviation of error are lower than those of the method of impedance control and the model-based reinforcement learning algorithm, the mean absolute errors of the force signal in the four groups are significantly reduced, further illustrating the strong stability of the proposed algorithm.

In the current work, we use constant force control, which is suitable for some scenarios of robot-skin contact, such as auxiliary treatment and robot local massage. In future research, we will study variable force to make the use range of the force controller wider.

## Data availability statement

The raw data supporting the conclusions of this article will be made available by the authors, without undue reservation.

## Ethics statement

The studies involving humans were approved by School of Mechanical and Engineering, Guangzhou University. The studies were conducted in accordance with the local legislation and institutional requirements. The participants provided their written informed consent to participate in this study.

## Author contributions

MX: Conceptualization, Methodology, Software, Validation, Writing—original draft, Writing—review & editing. XZ: Conceptualization, Methodology, Validation, Writing—original draft, Writing—review & editing. TZ: Conceptualization, Methodology, Writing—review & editing. SC: Conceptualization, Methodology, Software, Validation, Writing—review & editing. YZ: Methodology, Software, Writing—review & editing. WW: Funding acquisition, Methodology, Validation, Writing—original draft, Writing—review & editing.
